# Dynamics and Structure-Function Relationships of the Lamin B Receptor (LBR)

**DOI:** 10.1371/journal.pone.0169626

**Published:** 2017-01-24

**Authors:** Ioannis Giannios, Eleftheria Chatzantonaki, Spyros Georgatos

**Affiliations:** 1 Stem Cell and Chromatin Group, The Institute of Molecular Biology and Biotechnology, Biomedical Division, FORTH-ITE, Heraklion, Crete, Greece; 2 The Laboratory of Biology, The University of Ioannina, School of Medicine, Ioannina, Greece; University of Toronto, CANADA

## Abstract

The lamin B receptor (LBR) is a multi-spanning membrane protein of the inner nuclear membrane that is often employed as a “reporter” of nuclear envelope dynamics. We show here that the diffusional mobility of full-length LBR exhibits significant regional variation along the nuclear envelope, consistent with the existence of discrete LBR microdomains and the occurrence of multiple, asymmetrically-spaced anastomoses along the nuclear envelope-endoplasmic reticulum interface. Interestingly, a commonly used fusion protein that contains the amino-terminal region and the first transmembrane domain of LBR exhibits reduced mobility at the nuclear envelope, but behaves similarly to full-length LBR in the endoplasmic reticulum. On the other hand, carboxy-terminally truncated mutants that retain the first four transmembrane domains and a part or the whole of the amino-terminal region of LBR are generally hyper-mobile. These results suggest that LBR dynamics is structure and compartment specific. They also indicate that native LBR is probably “configured” by long-range interactions that involve the loops between adjacent transmembrane domains and parts of the amino-terminal region.

## Introduction

The lamin B receptor (LBR) is an integral protein of the inner nuclear membrane originally identified by *in vitro* binding to lamin B [1–3]. The LBR molecule contains eight (predicted) transmembrane domains. These domains exhibit high sequence homology to fungal and animal sterol reductases [4] and are flanked by two hydrophilic end-pieces at the amino- and carboxy-terminal ends [5–7]. The carboxy-terminal “tail” is rather short (32 residues) and does not contain recognizable structural features or motifs. However, the amino-terminal “head” region is quite large (> 200 residues) and consists of three distinct domains: the Tudor domain (Td); the serine-arginine (SR)-rich “hinge”; and the globular domain (GD) [8,9].

As all membrane proteins, LBR is synthesized on the surface of the endoplasmic reticulum (ER). After biosynthesis, the protein diffuses laterally across the ER, reaches the outer nuclear mebrane, passes through the nuclear pore complexes (NPCs) and arrives at the inner nuclear membrane, where is “trapped” by binding to nuclear lamina and peripheral heterochromatin [10–13]. The amino-terminal region of LBR, which contains specific binding sites for a variety of nuclear proteins and self-associates *in vitro* [3,6,8,9,14–19], is generally believed to contribute in nuclear retention. However, several studies have shown that also critical for correct targeting of LBR is the presence of at least one transmembrane domain –and preferably transmembrane I [5,20–23].

Based on these data, a GFP-fusion protein representing the amino-terminal region and the transmembrane domain I of LBR has been widely used as a “reporter” of nuclear envelope (NE) dynamics [12,13,17,24–27]. Such a “minimal construct” is often preferred, because elimination of (all but one of) the transmembrane domains seems to prevent the formation of inclusion bodies, which occurs with variable frequency when cultured cells are transfected with full-length LBR [24]. However, there is a flip-side to this “advantage”: it is now known that several mutations that map downstream to transmembrane domain I affect dramatically the function and cellular partitioning of LBR [28–34]; therefore, it is unlikely that carboxy-terminally truncated constructs, such as the one mentioned above, could faithfully report the dynamics of the native protein.

To investigate the structural determinants that are involved in LBR dynamics, we assessed the mobility of full-length and partially truncated proteins employing an array of quantitative and morphological approaches. The data reveal that LBR is part of multiple dynamic ensembles, which possess distinct diffusional properties and are distributed non-uniformly along the inner nuclear membrane. Despite this regional variability, comparison of ensemble-averaged data allows us to conclude that carboxy-terminal LBR mutants have distinct properties in relation to full-length LBR. Furthermore, the results suggest that oligomerization of LBR *in situ* and formation of “local structures” *via* intra-molecular interactions are probably critical for the correct partitioning of the protein along the inner nuclear membrane.

## Materials and Methods

### Cells

Hela, Hek293 (a generous gift from Dr. Frank Fackelmayer, ATCC No CRL-1573), MCF-7 (ATCC No HTB-22), NIH/3T3 (a generous gift from Dr. Carol Murphy, ATCC No CRL-1658), CaCO-2 (ATCC No HTB-37), C2C12 (Promochem ATCC No CRL-1772), CHO, COS (ATCC No CRL-1651) and U2OS (a generous gift from Dr. Frank Fackelmayer, ATCC No HTB-96) cell lines were cultured in Dulbecco’s Modified Eagle’s medium (DMEM) (Sigma Aldrich) supplemented with 2 mM L-glutamine, 2 mM penicillin/streptomycin and 10% fetal calf serum (FCS) (all from PAA) in a humidified incubator at 5% CO_2_ and 37°C. The MDCK cell line (a generous gift from Prof. Savvas Christoforidis, ATCC No CRL-2936) was cultured in DMEM supplemented with 2 mM L-glutamine, 2 mM penicillin/streptomycin and 10% FCS at 10% CO_2_ and 37°C. BHK cell line was cultured in DMEM supplemented with 2mM L-glutamine, 2mM penicillin/ streptomycin, 5% FCS and 10% tryptose phosphate (Gibco, Life Technologies).

### Constructs and transfection

The coding sequence of full-length mouse LBR was amplified using specific primers and mouse liver RNA as template. PCR fragments were then digested with EcoRI/BamHI and ligated in pEGFP-N2 (Clontech, Mountain View, CA). The rest of the constructs were generated by standard cloning procedures using as a template the FL-LBR and appropriate primers (see S1 Table). The Sec61β-eGFP construct was a generous gift of Dr. Carol Murphy (Univeristy of Birmingham, UK). All constructs were verified by sequence analysis. Transfection was carried out by PEI (Sigma Aldrich) according to manufacturer’s instructions, or by electroporation using the ECM630 apparatus (BTX) operated at 260 V, 85 μF, 725 Ω.

Mouse liver RNA was isolated with Trizol (1ml/50-100mg of tissue). The tissue was first frozen with liquid nitrogen and ground. Trizol was added, the specimen was incubated for 20 min at room temperature and centrifuged at 4°C. Then, the standard procedure of chloroform/isopropyl alcohol extraction was followed and the RNA pellet was washed with 75% EtOH. Finally, RNA was dissolved in DECP-H_2_O. The mouse used for the liver RNA isolation was not specifically breed for this study. Ethics Committee approval was not required per our Institution’s research regulations.

### Imaging

Cells grown on coverslips were washed, fixed in 4% formaldehyde in phosphate buffered saline, permeabilized with 0.2% Triton X-100 and blocked with 0.5% fish skin gelatin. The specimens were visualized in a Leica TCS SP5II confocal microscope. For multi-color analysis sequential image acquisition was applied and emission detection ranges were adjusted to minimize crosstalk between the different signals. The following antibodies were used: anti-Nup84/107, anti-BiP (obtained from Developmental Studies Hybridoma Bank); anti-lamin-B [2] (prepared and purified in the EMBL animal facility from rabbits not particularly breed for this study). Ethics Committee approval was not required per our Institution’s research regulations. For STED microscopy, Hela cells transfected with LBR-YFP were fixed in 4% formaldehyde in phosphate-buffered saline, permeabilized with 0.2% Triton X-100 and blocked with 0.5% fish skin gelatin. Specimens were examined under a 100x objective in a Leica SP5II STED microscope.

### Live imaging and FRAP/FLIP assays

FRAP experiments were performed in a Leica SP5 confocal microscope using a 63X /1.4 NA oil immersion objective and a 135 mW Argon/Neon laser. Cells were imaged in glass bottom dishes (MatTeK Corporation) kept at 37°C using an air-stream stage incubator. For membrane-spanning constructs, recovery data were acquired with the argon laser operating at 10% laser power and 15% transmission. The confocal pinhole was set to 3.99 Airy units. The zoom factor ranged from 7 to 10. Images were 512 X 512 pixels, line average was set at 2 and scan speed at 400 Hz. Prebleach images were collected at 6.5 s time intervals for 32 s. Photobleaching in the NE was performed using either a circle of 1.0 or 1.5 μm in diameter, or a strip 1.7 μm wide and 10–70 μm in contour length. Photobleaching in the ER was performed using a 3 μm-circle ROI. The bleach duration was 2.6 s, with laser power at maximum. Postbleach images were collected for ~ 307 s (6 images at 3.5 s time intervals, followed by 8 images at 35 s intervals). For “soluble” constructs, recovery data were acquired with the argon laser operating at 10% laser power and 15% transmission. The confocal pinhole was set to 6.6 Airy units. The zoom factor ranged from 10 to 13. Images were 128 X 128 pixels. Scanning was bidirectional with scan speed at 800 Hz. Ten prebleach images were acquired at 0.38 s time intervals, while postbleach images were collected at 0.18 s time intervals for ~ 12.6 s. The photobleach was performed in a circle ROI 3.0 μm in diameter (unless otherwise specified) with bleach duration 0.36 s and laser power at maximum. For FRAP analysis, data were corrected for fluorescence quench and recovery observed in the entire cell and in the background, using the following formulas:

$$\text{I}/{\text{I}}_{0}=\left({\text{I}}_{\text{t}}-{\text{I}}_{\text{first postbleach}}\right)/\left({\text{I}}_{\text{prebleach average}}-{\text{I}}_{\text{first postbleach}}\right)$$

and

$${\text{I}}_{\text{t}}=\left({\text{I}}_{\text{t frap}}-{\text{I}}_{\text{t backround}}\right)/\left({\text{I}}_{\text{t unfrap}}-{\text{I}}_{\text{t backround}}\right),$$

where the *t* subscript denotes the specific time point for which the intensity (*I*) is calculated and *unfrap* represents an area of the same size with the ROI positioned outside of the bleached region. We confirmed that multiple consecutive FRAP experiments in the same cell and ROI (pulse-FRAP) give similar results and that cell viability is not affected. For FLIP assays, the samples were bleached repetitively and fluorescence loss in the non-bleached region was monitored. FLIP experiments were performed with the same settings as that for FRAP.

### Statistical and quantitative analysis

The coefficient of variation (CV) was calculated as the ratio of the standard deviation to the mean in each dataset. The Kolmogorov-Smirnov test was applied using the BioStat 2009 software (AnalystSoft).

## Results

### Distribution and dynamic properties of full-length LBR

To analyze the dynamic properties of LBR, we engineered 16 eGFP-fusion proteins (all carboxy-terminally tagged) that correspond to full-length LBR (FL-LBR) or LBR mutants (S1A Fig). Experiments with several cell lines showed that over-expression of FL-LBR does not generally perturb cellular architecture (S1B Fig). However, in one particular line (osteosarcoma U2OS cells) accumulation of FL-LBR caused detachment of the outer from the inner nuclear membrane in ~30% of the cells, as previously reported by Zwerger and co-workers [34]. For this reason, we continued our experimental work using Hela cells as a model system.

**Fig 1:**
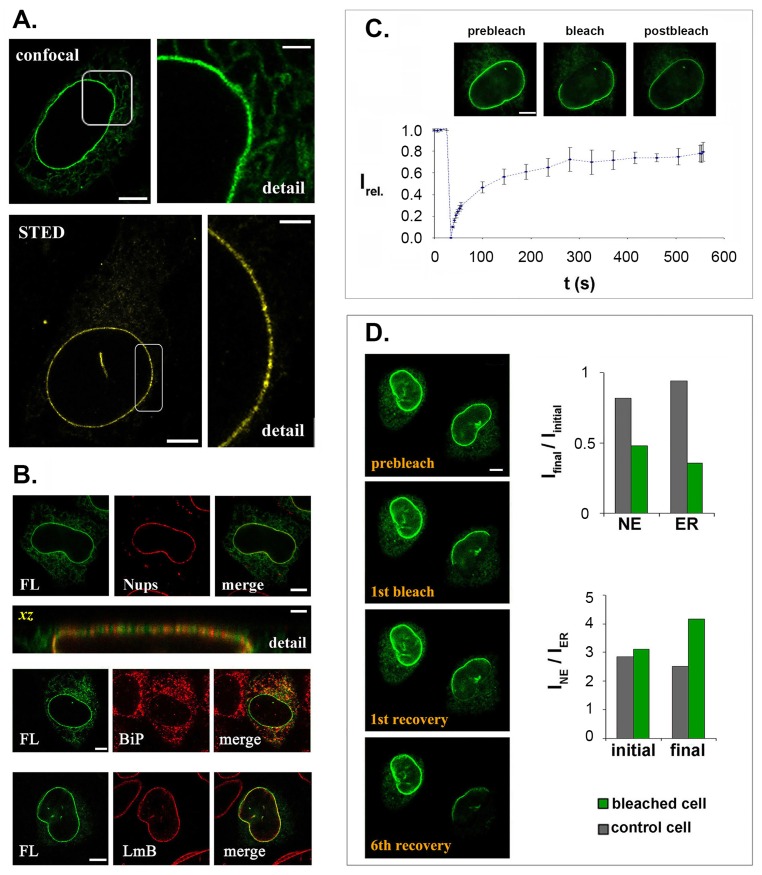
Localization and diffusional mobility of LBR in Hela cells. (A) Distribution of FL-LBR as detected by confocal and super-resolution (STED) light microscopy. The panels on the right show details in higher zoom and contrast. (B) Staining of FL-LBR expressing cells with antibodies against nucleoporins (Nup84/107), the ER protein BiP and nuclear lamin B (LmB). The distinct pattern of FL-LBR and Nups is more obvious in the xz section. (C) A typical FRAP assay with FL-LBR (the data represent an average of 3 independent experiments). *I*_*rel*_: normalized fluorescence intensity; *t*: time. (D) A representative FLIP experiment involving 6 successive bleaching-recovery cycles. The histograms depict the changes in fluorescence intensity (*I*_*final*_ */I*_*initial*_) at the NE and the ER. Bars in all panels are 6 μm and in “detail” panels 2 μm.

Upon transient transfection of Hela cells, FL-LBR fluorescence was detected in both the ER and the NE (Fig 1A). Based on intensity per unit area measurements, the fusion protein was ~3-fold more concentrated at the nuclear periphery than in the surrounding cytoplasm, consistent with retention in the inner nuclear membrane. FL-LBR co-localized to a large extent with nuclear lamin B, but its distribution pattern of was distinct from that of BiP (a luminal protein of the ER) and Nup84/107 (a marker of the NPC) (Fig 1B). In addition, the eGFP fusion protein formed closely spaced microdomains, similar to those identified in previous studies using specific antibodies [16,35]. These raft-like structures, which had contour lengths from 300–600 nm, were readily detectable by both conventional and super-resolution (STED) light microscopy (Fig 1A, *detail*).

To measure the diffusional mobility of FL-LBR, we took a FRAP/FLIP approach. In a typical FRAP experiment, fluorescence recovery at the NE reached a plateau 250–300 s after the end of the bleach pulse and remained steady for at least 500 s (Fig 1C). After normalization (see Materials and Methods), the ratio of the post-bleach to pre-bleach fluorescence intensity was consistently <1.0, indicating that a proportion of LBR is immobilized, presumably by binding to nuclear sub-structures. FLIP assays showed that non-bound FL-LBR molecules exchange throughout the ER-NE continuum: when a part of the NE was repeatedly bleached, a significant loss of fluorescence was detected in both the peripheral ER and the non-bleached sector of the NE (Fig 1D, *image series*). The loss of fluorescence at the NE was always less than the loss of fluorescence in the ER (Fig 1D, *histograms*), consistent with a higher mobility in the latter compartment.

**Fig 2:**
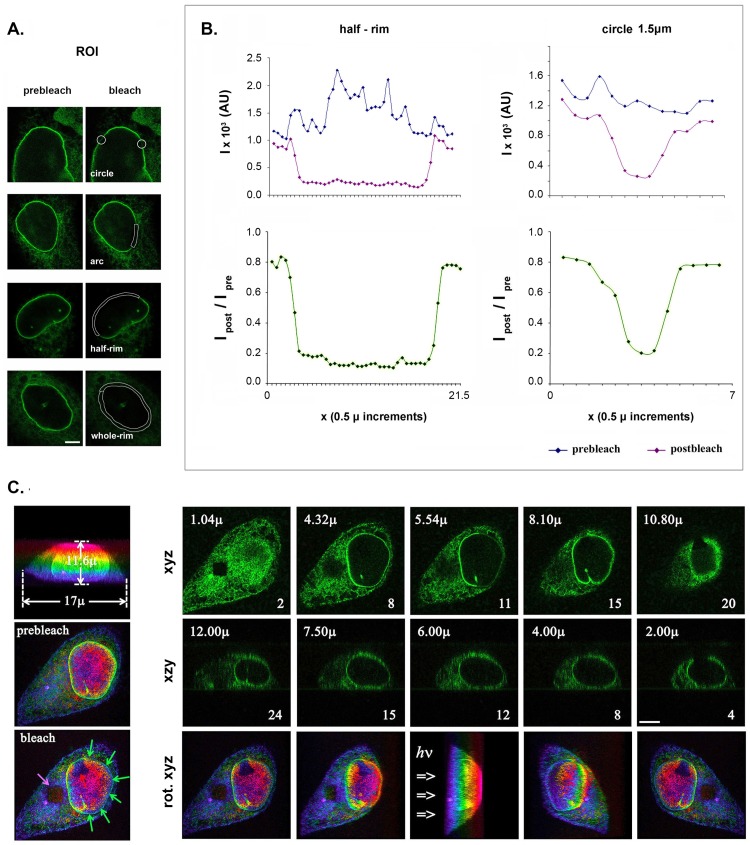
ROI geometries and bleach volume. (A) ROIs employed in the FRAP assays. The panels show FL-LBR distribution before and after bleaching different parts of the NE, as indicated in the images. Bar, 6 μm. (B) Intensity profiles before and after bleaching a circular spot with a diameter of 1.5 μm and a curvilinear strip (half-rim) in fixed Hela cells. Notice the nearly Gaussian profiles and the moderate “halo” effect (Δx ~1.0 μm). (C) Bleach depth after focusing the laser beam at the top (triangle, *pink*), equator (curvilinear segment, *green*), or the bottom (square, *violet*) of the cell. The series show successive optical sections at the *xy* and *xz* level, or rotated images (*rot*. *xyz*), revealing an axial bleach half-depth of 1.4–2.6 μm (distally and proximally to the beam, respectively). Pseudo-color according to sample depth was applied using the *LAS-AF* program.

To study LBR dynamics in more detail, we experimented with spot-like (circular) and strip-like (curvilinear) regions-of-interest (ROIs). The circular ROIs were 1.0 or 1.5 μm in diameter, while the curvilinear ROIs (referred to as “arc”, “half-rim” and “whole-rim”) were 1.7 μm in width and had contour lengths from 10–70 μm (Fig 2A). Photobleaching of fixed cells showed that the bleach profile across the ROIs was roughly Gaussian, with a lateral halo <1 μm (Fig 2B). However, with the optical setup employed (see *Experimental Procedures*), the bleached region had a substantial axial half-depth, extending about 2.6 μm proximally and 1.2 μm distally to the incoming beam (Fig 2C). Therefore, the *effective ROI* extended beyond the confines of the NE (which has a thickness of ~70 nm) and evidently included a part of the perinuclear ER.

**Fig 3:**
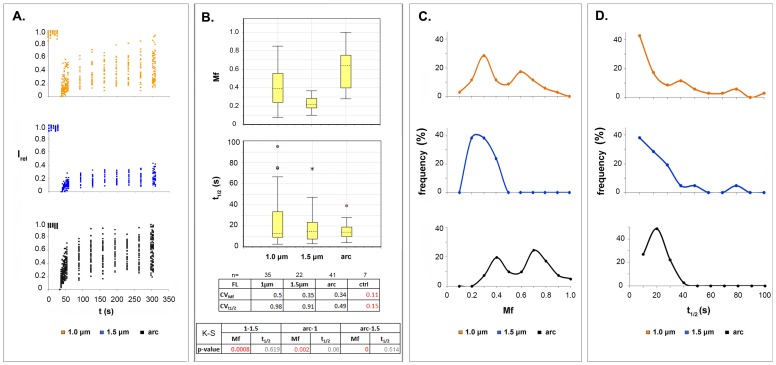
LBR mobility differences at the level of cell population. (A) FRAP assays using circular ROIs with diameters of 1.0 μm or 1.5 μm and an arc-shaped ROI approximately 1.7 μm wide and 10 μm long. (B) *Mf* and *t*_*1/2*_ box plots for all samples presented in (A). Statistical analysis based on the Kolmogorov-Smirnov test, sample number and coefficients of variation (*CV*) are specified. Red lettering indicates statistically significant differences with a threshold of 0.005. *CVctrl* indicates variation due to experimental error as determined in S2A Fig. (C) Frequency distribution of *Mf*. (D) Frequency distribution of *t*_*1/2*_.

Irrespective of ROI type used in the FRAP assays, the recovery curves of FL-LBR differed significantly from one experiment to the next, yielding a range of mobile fraction (*Mf*) and recovery halftime (*t*_*1/2*_) values (Fig 3A). To distinguish between genuine differences in protein mobility and fluctuations due to physical perturbation, we performed a series of control experiments. Potential flaws arising from photodamage or shifting of the cells during data acquisition were assessed recording fluorescence recovery after *consecutive* bleaching of the *same cell and ROI* (pulse-FRAP assays). As could be seen in S2A Fig, the coefficients of variation (*CVs*) of *Mf* and *t*_*1/2*_ in pulse-FRAP assays were significantly lower than that recorded by sampling different cells and ROIs. Furthermore, a comparison of the corresponding cumulative distribution functions (CDFs) by the Kolmogorov-Smirnov (K-S) test showed that the dynamic parameters of FL-LBR did not vary in a systematic fashion in relation to the pulse sequence. Finally, in cells whose FL-LBR content differed more than 3-fold, the corresponding *CVs* were almost the same (S2B Fig). Taken together, these data suggest that LBR dynamics exhibits genuine variability.

When we used circular ROIs, the ensemble-average *Mf* of FL-LBR, as well as the CV_*Mf*_, inversely correlated with ROI size (Fig 3B). Examining the frequency distributions of *Mf*, we noticed that assays with 1-μm ROIs yielded a major peak at low *Mf* values and a less prominent peak at higher *Mf* values (Fig 3C, *upper graph*). However, assays with 1.5-μm ROIs yielded a unimodal *Mf* distribution, with a broad peak at low *Mf* values (Fig 3C, *middle graph*). This explains the lower *Mf* and CV_*Mf*_ obtained in the latter case and strongly suggests that LBR displays length scale-dependent heterogeneity. Interestingly, the distribution of *t*_*1/2*_ was rather insensitive to changes of ROI size (Fig 3D; *upper and middle graphs*), which implies that LBR binding to underlying sub-structures is dominant over diffusion [for more details on “reaction-dominant” and “diffusion-dominant” processes see ref.[36]].

Experiments with arc-shaped ROIs gave a bimodal *Mf* distribution (as observed previously with small circular ROIs). However, in this case the high *Mf* peak was more pronounced than the low *Mf* peak (Fig 3C, *lower graph*), resulting in an increase of the average *Mf* (Fig 3B). Expanding the contour length of the arc-shaped ROI resulted in a gradual decrease of *Mf* (S3 Fig). However, this should be interpreted with caution, because the visual inspection of these samples revealed extensive bleaching of the ER throughout the cell, probably due to the higher angle of the incoming beam (check Fig 2A, *whole-rim configuration*), which apparently results in the depletion of the unbleached LBR pool. Irrespective of the technical limitations encountered with very large ROIs, the distinct shifts in *Mf* distribution when the change of ROI size (or shape) was within limits suggested a regional variability.

**Fig 4:**
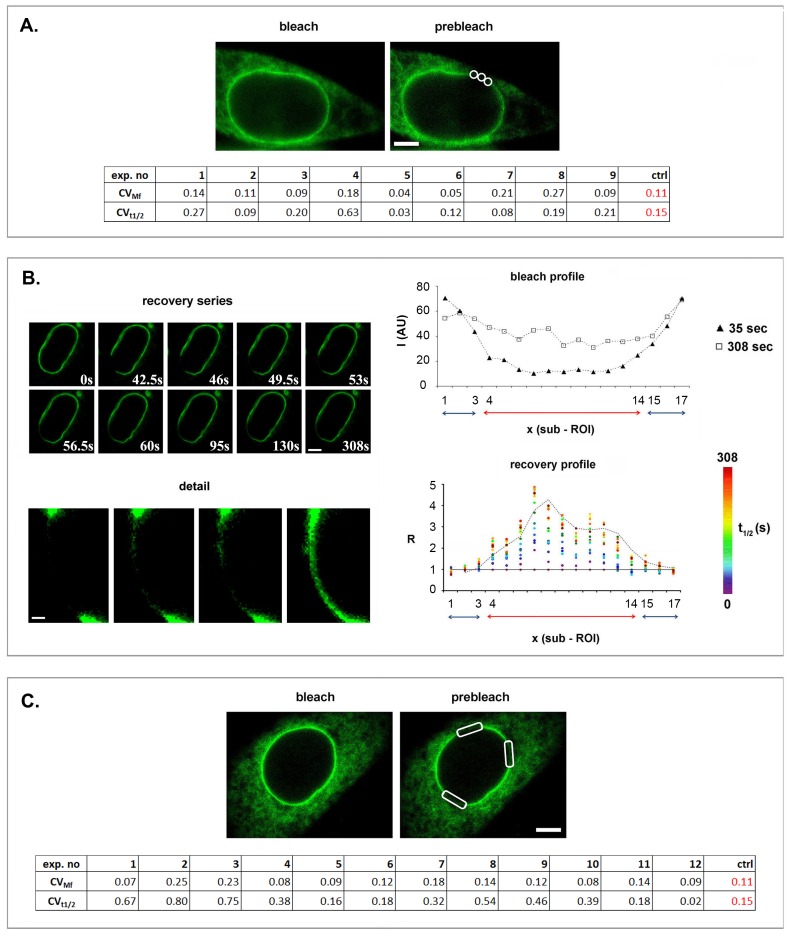
LBR mobility differences at the level of single cells. (A) FL-LBR recovery in the central area and the edges of the same arc-shaped ROI (see image). The table underneath shows the *CVs* for *Mf* and *t*_*1/2*_ in each experiment. The *CVs* of the control (*ctrl*), calculated as described in S2A Fig, are also included for a comparison. (B) A profile FRAP experiment with a sample exhibiting significant *Mf* and *t*_*1/2*_ variation across the same ROI. The upper panel depicts successive snapshots of the photobleached area at various time intervals. Details of the ROI at higher zoom and contrast are shown underneath. The bleach and recovery profiles along adjacent sub-ROIs (no. 1–17) are shown separately in the right panel. (*I*): fluorescence intensity; (*x*): contour length; (*R*): fluorescence intensity across successive sub-ROIs at various time intervals in relation to the fluorescence intensity immediately after the bleach. The bleach was done at *t* = 35 s and the recovery process was completed at *t* = 308 s. (C) Variation of LBR mobility in different sectors of the same NE (see images). The results are presented in the same format as in (*A*). Bars are 6 μm and 1 μm in (*B*) “detail”.

To examine more directly whether LBR dynamics exhibits such a regional variability, we performed experiments at the level of single cells. On a first approach, we asked whether FL-LBR recovers in a uniform fashion across *the same ROI*. In most of the samples, the mobility of FL-LBR at the edges and the central region of an arc-shaped ROI did not differ significantly (Fig 4A). However, in a few cases, the mobility of FL-LBR across the same ROI did differ, reaching the levels of variation that we had previously observed by assaying different cells and ROIs. To confirm that this variation reflected molecular events, we inspected frame-by-frame the recovery series in the corresponding samples and examined visually the re-distribution of fluorescence along the bleached zone. From this survey it became clear that FL-LBR recovery across a given ROI was asynchronous and non-uniform (Fig 4B; *image series*).

More detailed profile FRAP experiments showed that the non-uniformity of the recovery process was not due to uneven photobleaching across the ROI (Fig 4B; upper *graph*). Furthermore, when we measured the local rates of fluorescence recovery (*i*.*e*., the ratio of fluorescence intensity at a given time point divided by the fluorescence intensity immediately after bleaching), we noticed that the areas flanking the bleached region were gradually losing fluorescence as a function of time; conversely the central area of the ROI was gaining fluorescence (Fig 4B, *lower graph*). The rate of fluorescence recovery in the central zone of the ROI was not steady, displaying distinct “peaks” and “valleys”. Furthermore, the fluorescence intensity in different sub-regions did not always increase as a function of time, but sometimes regressed to a lower level. As a rule, this was paralleled by fluorescence gain in neighboring areas, revealing that segments of the NE that had transiently accumulated fluorescent molecules could act as a “source” for the flanking regions.

**Fig 5:**
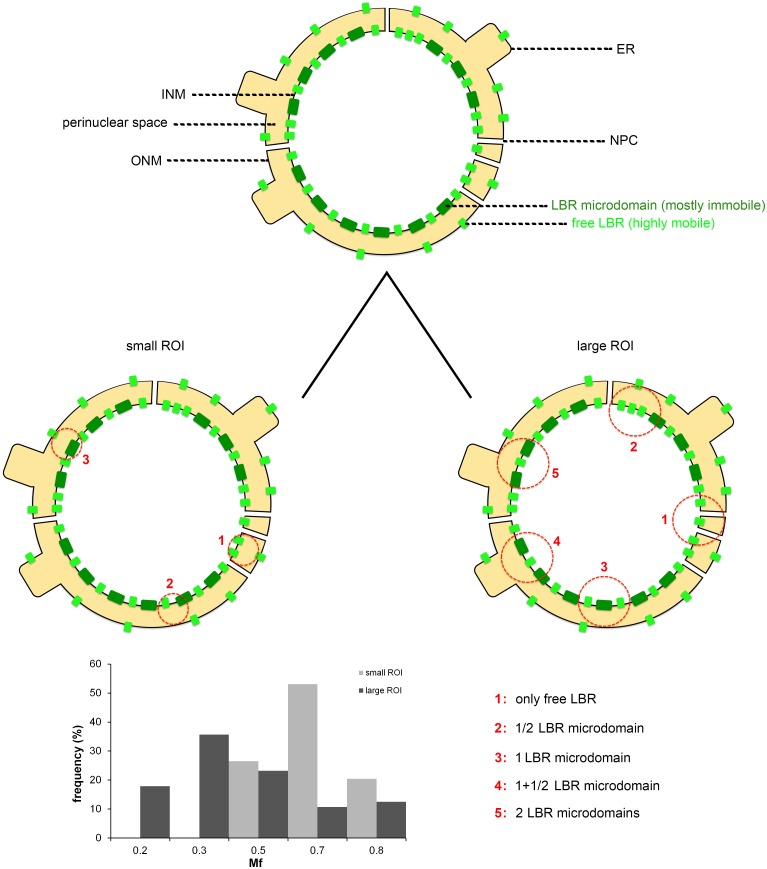
A provisional model explaining regional variation of LBR mobility. The “scanning probe” cartoon explains how LBR mobility could vary from one region of the NE to the next, depending on the relative abundance of immobile (*dark green*) or mobile (*light green*) LBR molecules. The basic assumptions in this model are that: a) ROI selection is entirely random and therefore, the probability of including or not including LBR microdomains is proportional to the abundance of the latter; b) LBR microdomains contain many more molecules than “free” LBR; c) the areas of the INM neighbouring to the NPCs, where the lamina meshwork is interrupted, contain exclusively free LBR. The same applies for the outer nuclear membrane and the surface of the ER. The histogram shown is based on an entirely hypothetical example, where the ROIs include from 0 to 2 microdomains each, depending on size. Arbitrarily, we have assumed that regions containing free LBR regain 80% of the initial fluorescence, whereas areas containing 0.5 to 2 microdomains regain from 20% to 70% of the fluorescence, respectively. Notice that increase of ROI size results in the broadening of the *Mf* frequency distribution (“homogenization”) and shifting of the average *Mf* towards lower values. ROIs are indicated by a circle. (*ER*): ER cisterna; (*ONM*): outer nuclear membrane; (*INM*): inner nuclear membrane; (*NPC*): nuclear pore complex. For further details see text.

When we probed non-contiguous sectors of the *same NE*, regional differences in the diffusional mobility of FL-LBR became even more apparent (Fig 4C). Based on these data, we would argue that variation of the dynamic parameters arises primarily from the non-uniform distribution of LBR along the inner nuclear membrane (i.e. the existence of discrete microdomains that contain predominantly immobile protein and stretches that contain mostly “free” LBR). Since ROI selection in the FRAP experiments is entirely random, it is likely that the area probed each time contains a variable number of LBR microdomains. Obviously, the more ROI size increases, the more probable it becomes that the bleached region will span both microdomain-rich and microdomain-poor regions of the NE. This explains the “homogenization effect” (lower variability) and the trend towards a lower *Mf* observed in the FRAP assays, as is graphically depicted in the “scanning model” of Fig 5.

We have shown above (Fig 2C), that cisternae of perinuclear ER are inescapably included in the bleach volume when the laser beam is focused on the surface of the nucleus, due to light diffraction. Knowing that ER resident proteins often exhibit variable dynamic behavior due to the spatial complexity of the endomembranes [37], we found it reasonable to compare the variation of LBR dynamics in the NE and the ER. As will be shown in the next section, the mobility of FL-LBR in the ER varied noticeably less than in the NE. However, in both compartments the dynamic parameters of FL-LBR fluctuated more than one would expect for a monodispersed protein species diffusing across the lipid bilayer. Therefore, at least a part of the variation observed in the FRAP assays should be attributed to the fact that LBR exists in many molecular forms and oligomeric states. This point should be taken seriously into account when pursuing a more definitive interpretation than that proposed in Fig 5.

### Dynamics of membrane-spanning and “soluble” LBR mutants

Along with FL-LBR, we also examined several forms of the protein that lack parts of the carboxy-terminal region (see S1A Fig). Among these were: Gr (or ΔTMVIII,CT), which lacks transmembrane domain VIII and resembles a pathogenic mutant associated with Greenberg’s dysplasia; Icj (or ΔTMV-VIII,CT), which lacks transmembrane domains V-VIII and resembles a mutant that causes mouse ichthyosis; and Mod (or ΔTMII-VIII,CT), which contains the entire amino-terminal and transmembrane domain I, resembling the “minimal construct” used in previous studies (see Introduction).

**Fig 6:**
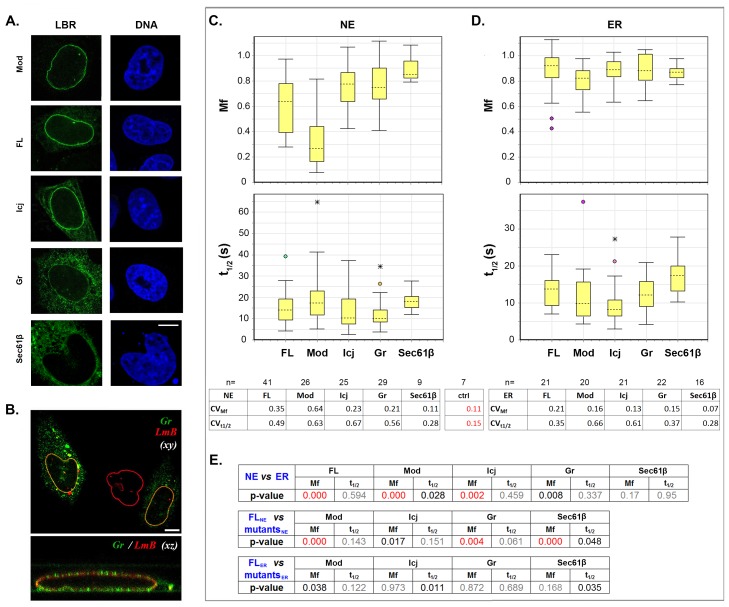
Properties of membrane-spanning LBR mutants. (A) Localization patterns of FL-LBR, LBR mutants and Sec61β (an ER membrane protein) after transfection of Hela cells. (B) Staining of Gr–expressing cells with anti-lamin B antibodies. Notice the cytoplasmic accumulations that stain positive for lamin Β in some of the cells. Bars in (A) and (B), 6 μm. (C-D) *Mf* and *t*_*1/2*_ box plots for all proteins using an arc-shaped ROI. The panel shows two separate sets of data obtained by probing either the NE or the peripheral ER. Sample number and coefficients of variation (*CVs*) are specified. *CVctrl* indicates variation due to experimental error as determined in S2A Fig. (E) Statistical evaluation and comparison of the data shown in (C-D) by the K-S test. The threshold of statistical significance is 0.005. Red lettering indicates significant differences; black lettering corresponds to differences close to the statistical threshold; and grey lettering denotes differences that are not significant.

The overall distribution of the three truncated proteins was similar to that of FL-LBR (Fig 6A). However, in about 20% of transfected cells, the Gr mutant over-accumulated in the cytoplasm and formed large clusters. Some of these clusters stained for lamin B, suggesting trapping of nuclear lamins by the ectopically localized protein (Fig 6B).

As shown in the previous experiments, FRAP assays with FL-LBR yielded variable readouts. The same was observed when we tested the three membrane-spanning LBR mutants (Fig 6C and 6D *box plots*). To find out whether the fluctuations of the dynamic parameters were protein or compartment-specific, we measured the mobility of FL-LBR and LBR mutants, together with Sec61β in the NE and the bulk ER. Sec61β is an integral membrane protein of the ER that also partitions (without being retained) with the NE [38]. As demonstrated in Fig 6C and 6D (*tables*), the *Mf* values of Sec61β exhibited variation close to the levels of experimental error in both compartments. On the other hand, whereas significant variation was detected at the NE with all LBR proteins (especially Mod), this did not happen in the ER. Variability in *t*_*1/2*_ was in all cases above the threshold of experimental error and most noticeable with the Mod and Icj mutants. From these results we infer that mobility fluctuations are both protein and compartment-specific, whereas fluctuations in exchange rate are only dependent on protein configuration.

Despite the variability, a comparison of the FRAP data in cells expressing intact or truncated forms of LBR could still be made comparing the corresponding *Mf* values by the K-S test (Fig 6E). Using this approach, we found that Icj and Mod were significantly more mobile in the ER than in the NE, as was FL-LBR. Gr behaved in roughly the same way, but the mobility differences in the two compartments were less clear-cut, consistent with the fact that this mutant over-accumulates in cytoplasmic membranes in a sub-population of the cells. By K-S criteria, the differences in exchange rate (*t*_*1/2*_) between NE-associated and ER-localized proteins were not significant. Taken together, these data suggest that the three LBR mutants are retained in the inner nuclear membrane by binding to underlying sub-structures, as does the native protein.

That said, it is important to note that Gr exhibited a higher mobility than FL-LBR at the NE, whereas Mod displayed lower mobility than the intact protein (Fig 6C). Icj also showed a higher mobility than FL-LBR, but this difference was not as significant by statististical criteria. The lower mobility of the Mod mutant and the increased mobility of Gr and Icj at the NE did not appear to be the consequences of structural aberrations that affect the diffusional properties of the protein, because none of the three mutants showed significant differences from FL-LBR in the bulk ER. Furthermore, the divergent properties of Mod and Gr rule out other effects resulting from the flipping of the hydrophilic carboxy-terminal tail of LBR from the nucleoplasm to the ER lumen. (According to the predicted topology of the LBR protein, in both of these mutants the tailpiece should be facing the lumenal side of the inner nuclear membrane, due to the fact that the truncated polypeptides contain an odd number of transmembrane domains; check S1A Fig)

To find out whether the lower mobility of Mod at the NE was due to the fact that the eGFP moiety is fused near transmembrane domain I (which might possess some unique structural properties), we did additional experiments. Another LBR mutant, bearing exactly the same amino-terminal part with Mod, but possessing transmembrane domain VI instead of transmembrane domain I at its carboxy-terminal region, was constructed and assyed by FRAP. As shown in S4B Fig, this mutant, termed NtTM4 (or ΔTMI-III,V-VIII,CT), exhibited similar properties to Mod at the NE, suggesting that the differences between FL-LBR and Mod are not due to the peculiar features of the first transmembrane domain. However, in the bulk ER, the NtTM4 mutant showed a faster exchange rate by comparison to FL-LBR. Taken together, these data suggest that carboxy-terminal truncations affect the dynamic properties of LBR in different ways, depending on the membrane compartment.

To get more insight, we also studied the properties of TL (or ΔCt), which lacks the entire hydrophilic tailpiece, and three more LBR mutants that contain transmembrane domain I (as the Mod protein), but miss various parts of the amino-terminal region (S1A Fig). No differences between any of these mutants and FL-LBR could be distinguished at the level of the confocal microscope (S4A Fig). However, when we measured their diffusional mobility by FRAP and analyzed the data as outlined above (S4B and S4C Fig and S4 Supplementary Text), we arrived at some interesting conclusions. First, whereas TL behaved similarly to FL-LBR, the mobility of some amino-terminally truncated mutants in the NE and the bulk ER did not differ. Thus, unlike the carboxy-terminal truncations described above, amino-terminal deletions could affect LBR retention in the inner nuclear membrane, presumably by interfering with binding to the nuclear lamina and the peripheral heterochromatin network. However, it should be taken into account that amino-terminally truncated mutants often behaved differently from FL-LBR in the ER, suggesting a more “global” effect on protein structure.

One point that should not be missed here is that the deletion of large segments from the LBR molecule did not render the corresponding mutants invariably hyper-mobile and faster exchanging. This is evident when one compares the properties of Mod to that of FL-LBR. On the other hand, it should be pointed out that the differences identified between FL-LBR and LBR mutants by applying statistical methods do not necessarily describe the properties of these proteins at the level of single molecules and single cells. As we have shown, individual assays sometimes yield large mobility differences, irrespective of which LBR form is expressed, due to the regional fluctuations of LBR dynamics.

**Fig 7:**
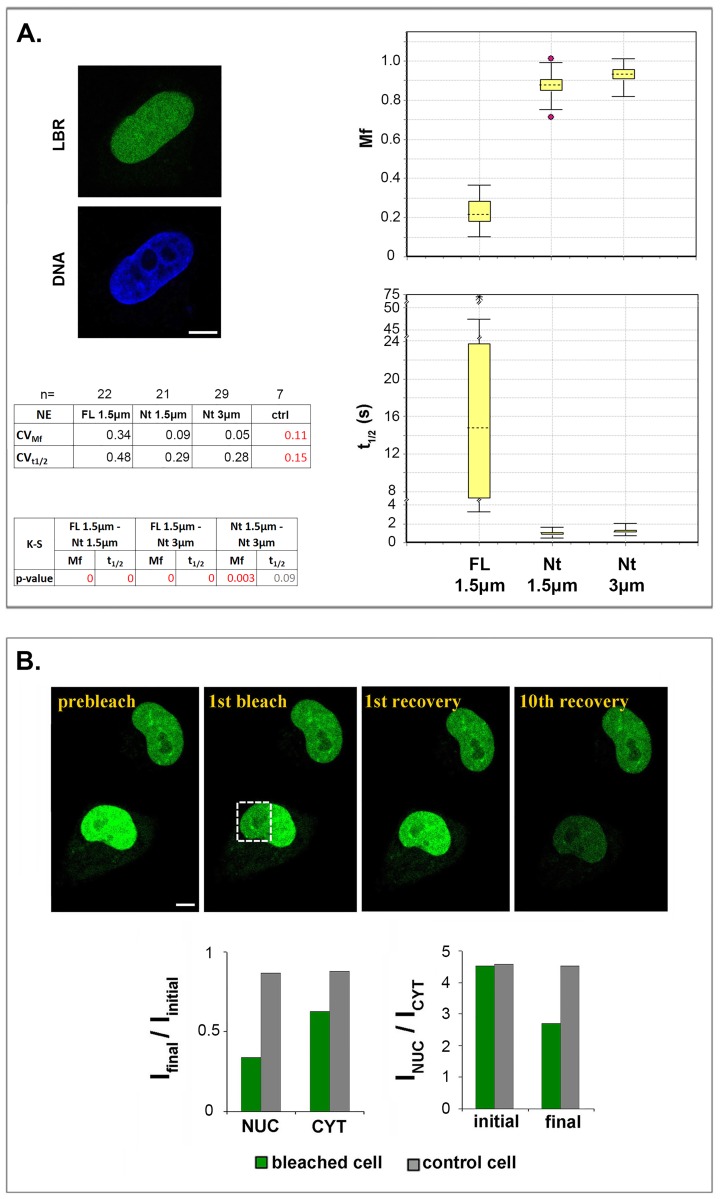
Properties of a “soluble” protein corresponding to the amino-terminal region of LBR. (A) Localization pattern and FRAP data obtained with the Nt mutant, after transfection of Hela cells. *Mf* and *t*_*1/2*_ box plots comparing the dynamic parameters of FL-LBR and Nt in FRAP assays with circular ROIs ranging from 1.5 to 3.0 μm in diameter. Statistical analysis based on the K-S test, sample number and coefficients of variation (*CVs*) are specified. *CVctrl* indicates variation due to experimental error as determined in S2A Fig. Notice that variation is much lower with Nt than with FL-LBR. (B) A typical FLIP experiment with Nt (10 successive bleaching-recovery cycles). The histograms show the changes in fluorescence intensity (*I*_*final*_ */I*_*initial*_) in the nucleoplasm (*NUC*) and the cytoplasm (*CYT*). Bars in all panels, 6 μm.

Proceeding further, we examined seven “soluble” mutants that do not contain any transmembrane domain. As shown in Fig 7A and S5A Fig, the soluble mutants did not accumulate at the nuclear periphery, confirming that the presence of a transmembrane domain is necessary for retention in this territory [for relevant observations see [5,20–23]]. Nevertheless, from these experiments it became clear that all mutants containing the RS domain accumulate in the nucleoplasm, while those lacking RS exhibit a more or less pan-cellular distribution, resembling that of free eGFP. This means that the RS motif is essential for translocation and retention of LBR into the cell nucleus.

Special care was taken to compare the properties of the soluble mutant Nt (or TDRSGD), which represents the entire amino-terminal region, to the properties of FL-LBR. When assessed at different ROI sizes, this mutant yielded much lower variation in *Mf* and *t*_*1/2*_ (Fig 7A), confirming that differences in dynamic parameters are specific to membrane-associated LBR.

Finally, FLIP assays with Nt revealed another aspect of LBR dynamics, which adds complexity to the diffusion-retention mechanism utilized to target the protein to the inner nuclear membrane. As shown in Fig 7B, the nucleoplasmically disposed Nt exchanged to a greater extent with nucleoplasmic than with cytoplasmic molecules. This indicates that NPCs impose a significant barrier that must be overcome before LBR molecules enter the cell nucleus [for further comments see [39]].

## Discussion

The introduction of “F-techniques” has revolutionized Cell Biology, allowing study of cellular events in real time. However, the widespread application of these methods has largely neglected the fact that, unlike model planar or spherical surfaces, real biological interfaces are rather anisotropic or have a complex, asymmetric architecture. The effect of surface geometry on the measurement of dynamic parameters cannot be overemphasized [40–42]. As it turns out, diffusion of integral membrane proteins is significantly different from diffusion of luminal or cytosolic components, with *t*_*1/2*_ varying by a factor of ~4, even when the membrane shapes are the same [37]. Furthermore, membrane geometry-induced variation in *t*_*1/2*_ can be as much as 1.8-fold [43].

The NE not only is a highly curved membrane, but its curvature changes from one region to the next. In addition, the number of ER “connections” along the outer nuclear membrane is highly variable, affecting the influx of membrane proteins from the ER to the NE at a local scale. Finally, the NE membranes are tightly associated with macromolecular assemblies (*e*.*g*., the nuclear lamina meshwork and peripheral heterochromatin), the spatial distribution of which is far from homogeneous. Considering these non-regularities, it is surprising that most of the studies conducted so far with NE proteins have treated this assembly as an “ideal” 2-D membrane surface and have not stumbled on quantitative differences that cannot be explained by mere experimental error.

To assess LBR dynamics, in this study we have taken an unbiased FRAP/FLIP approach. The choice of this approach was dictated by several factors. First, FRAP was precisely the method utilized in all previous studies on LBR dynamics, allowing us to compare our results with those reported by other authors. Second, FRAP permitted quantitative assessment of molecular mobility at good spatial resolution, which was a critical factor when assessing the properties of “fine” interfaces, such as the NE. Finally, FRAP assays were not particularly time consuming and could be used as a screening means for a large number of samples, as required.

FRAP does have several limitations and is not immune to artifacts [44,45]. Knowing that, we have made no presumptions on bleaching format and tried to be as consistent in sampling as we technically could. To supplement the data obtained by FRAP, we also considered other techniques, such as FCS. However, this technique proved rather problematic, because the very low fluorescence intensities required when applying it did not allow sufficiently good imaging and proper selection of ROI. Given the non-homogeneous distribution of LBR, future experiments based on single-particle tracking may provide more clues as to whether or not LBR microdomains represent “obstacles” that create a regime of anomalous diffusion at the NE and thus affect the dynamics of both LBR and other integral proteins.

Under our experimental conditions, significant variation was observed when LBR dynamics was assessed by conventional FRAP assays. In this context, the *Mf* of LBR in single nuclei varied up to 9-fold, while the *t*_*1/2*_ differed up to 40-fold, depending on which region and sub-region of the NE was probed. The mere magnitude of these differences strongly suggests that, beyond the known asymmetries, LBR itself probably exists in various binding states, transition intermediates and aggregation forms. Deriving specific thermodynamic and kinetic parameters from such a complex mode of dynamics is not realistic at the present time, because crucial details remain to be investigated. For instance, LBR has been reported to bind multiple proteins [1,6,14,46], is post-translationally modified at several sites [47] and follows a rather convoluted route from the peripheral ER to the inner nuclear membrane, after crossing the straights of the NPCs [39]. Without knowing which of these interactions and modifications are mutually exclusive and which prevail in a given micro-environment, it is simply not feasible to fit the experimental data to any of the current reaction-diffusion schemes. However, until we arrive at that point, useful information can be obtained by taking the FRAP data for what they worth. For example, evaluating the CDFs of the *Mf* and *t*_*1/2*_, one can compare the ensemble-average properties of different LBR forms and obtain valuable information as to whether or not specific mutations affect LBR dynamics and result in functional defects. A demonstration of this treatment is provided by the comparison we have made assessing the relative mobility of FL-LBR and Mod. From this assessment, it is clear that the mobility of the mutant protein in the NE is markedly lower than that of wild type LBR.

The lower mobility of Mod is contrasted by the apparent hyper-mobility of less radically truncated mutants, such as Gr and Icj, which are much shorter in amino acid sequence. This probably means that there are extensive intra-molecular interactions among the loop segments that connect the transmembrane domains to each other. Analogous interactions, which confer structural stability and ligand specificity in multi-spanning membrane proteins, have been identified over the years in several receptors and ion channels. With that as a given, it would not be wise to extrapolate directly from the linear sequence and the domain structure of LBR to the properties of truncated LBR, in an attempt to interpret their *in vivo* behaviour by a “loss-of-function” approach logic.

## Supporting Information

S1 Fig LBR constructs and cell lines tested in this study. (A) Schematic diagram of LBR mutants. *Td*: amino-terminal Tudor domain; *RS*: serine-arginine rich region; *GD*: globular domain; *I-VIII*: transmembrane domains; *Ct*: carboxy-terminal tail. The amino acids at the borders of different domains are indicated in blue. The sites used for engineering the main mutants are in red. All mutants are termed using a standard format that indicates which domains have been cut and which remain. However, to simplify the reading of the text, the key mutants presented in the main figures are also referred to as Icj, Gr and Mod. (B) FL-LBR distribution in different cell lines (apart from the Hela cells that are presented in the main figures). Note that expression of the protein in U2OS cells causes major NE defects (arrow), as described previously by Zwerger et al. [34], whereas expression in all other lines has no visible effect. Bar, 6 μm.
(TIF)

S2 Fig Control experiments. (A) Pulse-FRAP assays using an arc-shaped ROI configuration. The scatterplot shows the values of *Mf* and *t*_*1/2*_ in 7 independent experiments, where the same region of the NE was bleached and allowed to recover 2 times in a row with a 15-min interval in-between. The histograms underneath depict the *CVs* for *Mf* and *t*_*1/2*_. The first pulse in each sequence is represented by red symbols and the second by blue symbols. Grey symbols in the background correspond to the data shown in Fig 3 and are included for a comparison. The red (broken) line corresponds to the average *CV* calculated from the data shown in Fig 3; the green (broken) line corresponds to the average *CV* in the experiments presented here. The p-values of the K-S test (first versus second pulse) are indicated. (B) Protein mobility in cells expressing low and high levels of FL-LBR (see images; bar 6 μm). *Mf* and *t*_*1/2*_ box plots for all samples are presented. Statistical analysis using the K-S test, sample number and coefficients of variation (*CVs*) are specified. *CVctrl* indicates variation due to experimental error as determined in S2A Fig.
(TIF)

S3 Fig LBR mobility differences at the level of cell population, using arc-shaped ROIs. *Mf* box plot derived from FRAP assays using an arc-shaped ROI approximately 1.7 μm wide and 10 μm long, half-rim and whole-rim ROIs.
(TIF)

S4 Fig Properties of additional membrane-spanning LBR mutants. (A) Localization patterns of LBR mutants after transfection of Hela cells. Bars, 6 μm. (B-C) *Mf* and *t*_*1/2*_ box plots for all proteins using an arc-shaped ROI. The panel shows two separate sets of data obtained by probing either the NE or the peripheral ER. Sample number and coefficients of variation (*CVs*) are specified. *CVctrl* indicates variation due to experimental error as determined in S2A Fig. (D) Statistical evaluation and comparison of the data shown in (B-C) by the K-S test.
DESCRIPTIVE TEXT: **Dynamics of internally deleted and truncated LBR mutants**
To better understand the structure function relationships of LBR, apart from the mutants presented in the main text, we also examined the following mutants (S1A Fig): TL (missing the hydrophilic tailpiece); NtTM4 (missing the tailpiece and transmembrane domains I-III, V-VIII); ΔTd (missing the amino-terminal Tudor domain); TdRSTM1 (missing the tailpiece, the amino-terminal GD domain and transmembrane domains II-VIII) and TdTM1 (missing the amino-terminal RS and GD domains, as well as all sequences downstream to the transmembrane domain I).
The subcellular distribution of these mutants was similar to that of FL-LBR (S4A Fig). However, when we assayed their diffusional mobility by FRAP and analyzed the data by the K-S test (S4D Fig), we arrived at some interesting conclusions. First, no statistically significant differences were found when we compared the mobility and diffusion rate of ΔTd and TdRSTMI in the NE and the bulk ER. This suggested that, unlike carboxy-terminal truncations, amino-terminal truncations affect severely the ability of LBR to bind to underlying sub-structure. Second, the TdRSTMI and the TdTMI mutants appeared to be more mobile and faster exchanging than FL-LBR at the NE. However, this was less apparent with the ΔTd mutant, although this (minimally truncated) protein was generally as mobile at the NE as was in the bulk ER (see above). When we attempted a similar comparison with the ER-distributed mutants, we did not find differences between TdRSTMI and ΔTD, but TdTMI and ΔCt behaved differently in terms of mobility and exchange rate. From these data we infer that although amino-terminal truncations affect primarily LBR binding to the nuclear acceptor sites, the different mutant proteins do not diffuse with the same ease along the membrane.
(TIF)

S5 Fig Properties of additional “soluble” LBR mutants. (A) Representative profiles of Hela cells expressing LBR mutants that do not possess a transmembrane domain (see S1A Fig). (B) Corresponding *Mf* and *t*_*1/2*_ box plots after assaying the mobility of the soluble mutants in the cytoplasm (*CYT*) or the nucleoplasm (*NUC*). Sample number and coefficients of variation (*CVs*) are specified. *CVctrl* indicates variation due to experimental error as determined in S2A Fig.
(TIF)

S1 Table Primers used for the generation of LBR truncated constructs. (DOCX)

S1 Text Dynamics of internally deleted and truncated LBR mutants. (DOCX)

## References

[1] Worman HJ, Yuan J, Blobel G, Georgatos SD. A lamin B receptor in the nuclear envelope. Proc Natl Acad Sci U S A. 1988;85: 8531–4. Available: http://www.pubmedcentral.nih.gov/articlerender.fcgi?artid=282492&tool=pmcentrez&rendertype=abstract 2847165 10.1073/pnas.85.22.8531 PMC282492

[2] Simos G, Georgatos SD. The inner nuclear membrane protein p58 associates in vivo with a p58 kinase and the nuclear lamins. EMBO J. 1992;11: 4027–36. Available: http://www.pubmedcentral.nih.gov/articlerender.fcgi?artid=556913&tool=pmcentrez&rendertype=abstract 1327755 10.1002/j.1460-2075.1992.tb05496.x PMC556913

[3] Ye Q, Worman HJ. Primary structure analysis and lamin B and DNA binding of human LBR, an integral protein of the nuclear envelope inner membrane. J Biol Chem. 1994;269: 11306–11. Available: http://www.ncbi.nlm.nih.gov/pubmed/8157662 8157662

[4] Holmer L, Pezhman a, Worman HJ. The human lamin B receptor/sterol reductase multigene family. Genomics. 1998;54: 469–76. 10.1006/geno.1998.5615 9878250

[5] Worman HJ, Evans CD, Blobel G. The lamin B receptor of the nuclear envelope inner membrane: a polytopic protein with eight potential transmembrane domains. J Cell Biol. 1990;111: 1535–42. Available: http://www.pubmedcentral.nih.gov/articlerender.fcgi?artid=2116249&tool=pmcentrez&rendertype=abstract 2170422 10.1083/jcb.111.4.1535 PMC2116249

[6] Polioudaki H, Kourmouli N, Drosou V, Bakou a, Theodoropoulos P a, Singh PB, et al Histones H3/H4 form a tight complex with the inner nuclear membrane protein LBR and heterochromatin protein 1. EMBO Rep. 2001;2: 920–5. 10.1093/embo-reports/kve199 11571267 PMC1084077

[7] Holmer L, Worman HJ. Inner nuclear membrane proteins: functions and targeting. Cell Mol Life Sci. 2001;58: 1741–7. Available: http://www.ncbi.nlm.nih.gov/pubmed/11766875 10.1007/PL00000813 11766875 PMC11337314

[8] Ye Q, Callebaut I, Pezhman a, Courvalin JC, Worman HJ. Domain-specific interactions of human HP1-type chromodomain proteins and inner nuclear membrane protein LBR. J Biol Chem. 1997;272: 14983–9. Available: http://www.ncbi.nlm.nih.gov/pubmed/9169472 9169472 10.1074/jbc.272.23.14983

[9] Liokatis S, Edlich C, Soupsana K, Giannios I, Panagiotidou P, Tripsianes K, et al Solution structure and molecular interactions of lamin B receptor Tudor domain. J Biol Chem. 2012;287: 1032–42. 10.1074/jbc.M111.281303 22052904 PMC3256899

[10] Kourmouli N, Theodoropoulos P a, Dialynas G, Bakou A, Politou a S, Cowell IG, et al Dynamic associations of heterochromatin protein 1 with the nuclear envelope. EMBO J. 2000;19: 6558–68. 10.1093/emboj/19.23.6558 11101528 PMC305850

[11] Zuleger N, Kelly DA, Richardson AC, Kerr ARW, Goldberg MW, Goryachev AB, et al System analysis shows distinct mechanisms and common principles of nuclear envelope protein dynamics. J Cell Biol. 2011;193: 109–23. 10.1083/jcb.201009068 21444689 PMC3082195

[12] Boni A, Politi AZ, Strnad P, Xiang W, Hossain MJ, Ellenberg J. Live imaging and modeling of inner nuclear membrane targeting reveals its molecular requirements in mammalian cells. J Cell Biol. 2015;209: 705–720. 10.1083/jcb.201409133 26056140 PMC4460149

[13] Ungricht R, Klann M, Horvath P, Kutay U. Diffusion and retention are major determinants of protein targeting to the inner nuclear membrane. J Cell Biol. 2015;209: 687–704. 10.1083/jcb.201409127 26056139 PMC4460150

[14] Ye Q, Worman HJ. Interaction between an integral protein of the nuclear envelope inner membrane and human chromodomain proteins homologous to Drosophila HP1. J Biol Chem. 1996;271: 14653–6. 8663349 10.1074/jbc.271.25.14653

[15] Lin F, Noyer CM, Ye Q, Courvalin JC, Worman HJ. Autoantibodies from patients with primary biliary cirrhosis recognize a region within the nucleoplasmic domain of inner nuclear membrane protein LBR. Hepatology. 1996;23: 57–61. 10.1002/hep.510230109 8550049

[16] Makatsori D, Kourmouli N, Polioudaki H, Shultz LD, McLean K, Theodoropoulos P a, et al The inner nuclear membrane protein lamin B receptor forms distinct microdomains and links epigenetically marked chromatin to the nuclear envelope. J Biol Chem. 2004;279: 25567–73. 10.1074/jbc.M313606200 15056654

[17] Lu X, Shi Y, Lu Q, Ma Y, Luo J, Wang Q, et al Requirement for lamin B receptor and its regulation by importin {beta} and phosphorylation in nuclear envelope assembly during mitotic exit. J Biol Chem. 2010;285: 33281–93. 10.1074/jbc.M110.102368 20576617 PMC2963407

[18] Martins SB, Eide T, Steen RL, Jahnsen T, Skålhegg B S, Collas P. HA95 is a protein of the chromatin and nuclear matrix regulating nuclear envelope dynamics. J Cell Sci. 2000;113 Pt 21: 3703–13. Available: http://www.ncbi.nlm.nih.gov/pubmed/11034899 11034899 10.1242/jcs.113.21.3703

[19] Guarda A, Bolognese F, Bonapace IM, Badaracco G. Interaction between the inner nuclear membrane lamin B receptor and the heterochromatic methyl binding protein, MeCP2. Exp Cell Res. Elsevier Inc.; 2009;315: 1895–903. 10.1016/j.yexcr.2009.01.019 19331822

[20] Soullam B, Worman HJ. The amino-terminal domain of the lamin B receptor is a nuclear envelope targeting signal. J Cell Biol. 1993;120: 1093–100. Available: http://www.pubmedcentral.nih.gov/articlerender.fcgi?artid=2119726&tool=pmcentrez&rendertype=abstract 7679672 10.1083/jcb.120.5.1093 PMC2119726

[21] Soullam B, Worman HJ. Signals and structural features involved in integral membrane protein targeting to the inner nuclear membrane. J Cell Biol. 1995;130: 15–27. Available: http://www.pubmedcentral.nih.gov/articlerender.fcgi?artid=2120512&tool=pmcentrez&rendertype=abstract 7790369 10.1083/jcb.130.1.15 PMC2120512

[22] Smith S, Blobel G. The first membrane spanning region of the lamin B receptor is sufficient for sorting to the inner nuclear membrane. J Cell Biol. 1993;120: 631–7. Available: http://www.pubmedcentral.nih.gov/articlerender.fcgi?artid=2119546&tool=pmcentrez&rendertype=abstract 8381121 10.1083/jcb.120.3.631 PMC2119546

[23] Irons SL, Evans DE, Brandizzi F. The first 238 amino acids of the human lamin B receptor are targeted to the nuclear envelope in plants. J Exp Bot. 2003;54: 943–950. 12598565 10.1093/jxb/erg102

[24] Ellenberg J, Siggia ED, Moreira JE, Smith CL, Presley JF, Worman HJ, et al Nuclear membrane dynamics and reassembly in living cells: targeting of an inner nuclear membrane protein in interphase and mitosis. J Cell Biol. 1997;138: 1193–206. Available: http://www.pubmedcentral.nih.gov/articlerender.fcgi?artid=2132565&tool=pmcentrez&rendertype=abstract 9298976 10.1083/jcb.138.6.1193 PMC2132565

[25] Scott ES, O’Hare P. Fate of the inner nuclear membrane protein lamin B receptor and nuclear lamins in herpes simplex virus type 1 infection. J Virol. 2001;75: 8818–30. Available: http://www.pubmedcentral.nih.gov/articlerender.fcgi?artid=115126&tool=pmcentrez&rendertype=abstract 10.1128/JVI.75.18.8818-8830.2001 11507226 PMC115126

[26] Ostlund C, Sullivan T, Stewart CL, Worman HJ. Dependence of diffusional mobility of integral inner nuclear membrane proteins on A-type lamins. Biochemistry. 2006;45: 1374–82. 10.1021/bi052156n 16445279 PMC2527696

[27] Graumann K, Irons SL, Runions J, Evans DE. Retention and mobility of the mammalian lamin B receptor in the plant nuclear envelope. Biol Cell. 2007;99: 553–62. Available: http://www.ncbi.nlm.nih.gov/pubmed/17868028 17868028 10.1042/bc20070033

[28] Hoffmann K, Dreger CK, Olins AL, Olins DE, Shultz LD, Lucke B, et al Mutations in the gene encoding the lamin B receptor produce an altered nuclear morphology in granulocytes (Pelger-Huët anomaly). Nat Genet. 2002;31: 410–4. 10.1038/ng925 12118250

[29] Best S, Salvati F, Kallo J, Garner C, Height S, Thein SL, et al Lamin B-receptor mutations in Pelger-Huët anomaly. Br J Haematol. 2003;123: 542–4. Available: http://www.ncbi.nlm.nih.gov/pubmed/14617022 14617022 10.1046/j.1365-2141.2003.04621.x

[30] Oosterwijk JC, Mansour S, van Noort G, Waterham HR, Hall CM, Hennekam RCM. Congenital abnormalities reported in Pelger-Huët homozygosity as compared to Greenberg/HEM dysplasia: highly variable expression of allelic phenotypes. J Med Genet. 2003;40: 937–941. 10.1136/jmg.40.12.937 14684694 PMC1735340

[31] Waterham HR, Koster J, Mooyer P, Noort Gv G Van, Kelley RI, Wilcox WR, et al Autosomal recessive HEM/Greenberg skeletal dysplasia is caused by 3 beta-hydroxysterol delta 14-reductase deficiency due to mutations in the lamin B receptor gene. Am J Hum Genet. 2003;72: 1013–7. Available: http://www.pubmedcentral.nih.gov/articlerender.fcgi?artid=1180330&tool=pmcentrez&rendertype=abstract 12618959 10.1086/373938 PMC1180330

[32] Shultz LD, Lyons BL, Burzenski LM, Gott B, Samuels R, Schweitzer P a, et al Mutations at the mouse ichthyosis locus are within the lamin B receptor gene: a single gene model for human Pelger-Huët anomaly. Hum Mol Genet. 2003;12: 61–9. Available: http://www.ncbi.nlm.nih.gov/pubmed/12490533 12490533 10.1093/hmg/ddg003

[33] Gaudy-Marqueste C, Roll P, Esteves-Vieira V, Weiller P-J, Grob JJ, Cau P, et al LBR mutation and nuclear envelope defects in a patient affected with Reynolds syndrome. J Med Genet. 2010;47: 361–70. 10.1136/jmg.2009.071696 20522425

[34] Zwerger M, Kolb T, Richter K, Karakesisoglou I, Herrmann H. Induction of a massive endoplasmic reticulum and perinuclear space expansion by expression of lamin B receptor mutants and the related sterol reductases TM7SF2 and DHCR7. Mol Biol Cell. 2010;21: 354–68. 10.1091/mbc.E09-08-0739 19940018 PMC2808238

[35] Chmielewska M, Dubińska-Magiera M, Sopel M, Rzepecka D, Hutchison CJ, Goldberg MW, et al Embryonic and adult isoforms of XLAP2 form microdomains associated with chromatin and the nuclear envelope. Cell Tissue Res. 2011;344: 97–110. 10.1007/s00441-011-1129-2 21347574 PMC3112025

[36] Sprague BL, McNally JG. FRAP analysis of binding: proper and fitting. Trends Cell Biol. 2005;15: 84–91. 10.1016/j.tcb.2004.12.001 15695095

[37] Sbalzarini IF, Hayer A, Helenius A, Koumoutsakos P. Simulations of (an)isotropic diffusion on curved biological surfaces. Biophys J. 2006;90: 878–85. 10.1529/biophysj.105.073809 16284262 PMC1367112

[38] Wang Y, Yamaguchi H, Huo L, Du Y, Lee H, Lee H, et al The Translocon Sec61β Localized in the Inner Nuclear Membrane Transports Membrane-embedded EGF Receptor to the Nucleus. 2010;285: 38720–38729. 10.1074/jbc.M110.158659 PMC2992305 20937808

[39] Lusk CP, Blobel G, King MC. Highway to the inner nuclear membrane: rules for the road. Nat Rev Mol Cell Biol. 2007;8: 414–20. 10.1038/nrm2165 17440484

[40] Feder TJ, Brust-Mascher I, Slattery JP, Baird B, Webb WW. Constrained diffusion or immobile fraction on cell surfaces: a new interpretation. Biophys J. 1996;70: 2767–73. 10.1016/S0006-3495(96)79846-6 8744314 PMC1225256

[41] Owen DM, Williamson D, Rentero C, Gaus K. Quantitative microscopy: protein dynamics and membrane organisation. Traffic. 2009;10: 962–71. 10.1111/j.1600-0854.2009.00908.x 19416480

[42] Rayan G, Guet J-E, Taulier N, Pincet F, Urbach W. Recent applications of fluorescence recovery after photobleaching (FRAP) to membrane bio-macromolecules. Sensors (Basel). 2010;10: 5927–48. 22219695 10.3390/s100605927 PMC3247740

[43] Tian a, Baumgart T. Sorting of lipids and proteins in membrane curvature gradients. Biophys J. Biophysical Society; 2009;96: 2676–88. 10.1016/j.bpj.2008.11.067 PMC2711293 19348750

[44] Mueller F, Wach P, McNally JG. Evidence for a common mode of transcription factor interaction with chromatin as revealed by improved quantitative fluorescence recovery after photobleaching. Biophys J. 2008;94: 3323–39. 10.1529/biophysj.107.123182 18199661 PMC2275703

[45] Bates IR, Wiseman PW, Hanrahan JW. Investigating membrane protein dynamics in living cells. Biochem Cell Biol. 2006;84: 825–31. 10.1139/o06-189 17215870

[46] Clever M, Funakoshi T, Mimura Y, Takagi M, Imamoto N. The nucleoporin ELYS/Mel28 regulates nuclear envelope subdomain formation in HeLa cells. Nucleus. 2012;3: 187–99. 10.4161/nucl.19595 22555603 PMC3383574

[47] Nikolakaki E, Simos G, Georgatos SD, Giannakouros T. A nuclear envelope-associated kinase phosphorylates arginine-serine motifs and modulates interactions between the lamin B receptor and other nuclear proteins. J Biol Chem. 1996;271: 8365–72. 8626534 10.1074/jbc.271.14.8365

