# Supplementary figures and images for: Dynamics and Structure-Function Relationships of the Lamin B Receptor (LBR)
Source: PLoS One. 2017 Jan 24;12(1):e0169626. doi: 10.1371/journal.pone.0169626 (PMC5261809; doi:10.1371/journal.pone.0169626)

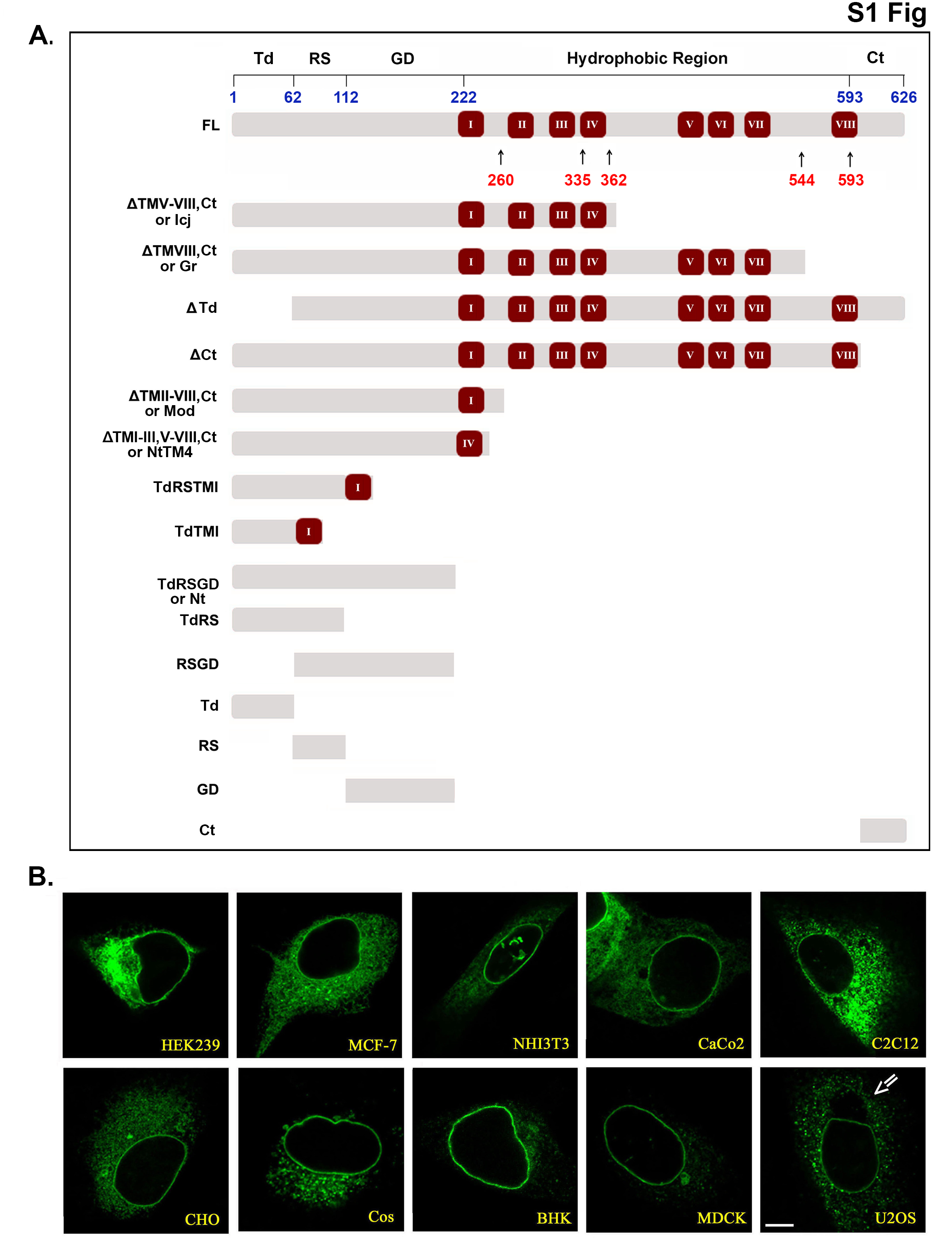

Supplement: S1 Fig — (A) Schematic diagram of LBR mutants. Td: amino-terminal Tudor domain; RS: serine-arginine rich region; GD: globular domain; I-VIII: transmembrane domains; Ct: carboxy-terminal tail. The amino acids at the borders of different domains are indicated in blue. The sites used for engineering the main mutants are in red. All mutants are termed using a standard format that indicates which domains have been cut and which remain. However, to simplify the reading of the text, the key mutants presented in the main figures are also referred to as Icj, Gr and Mod. (B) FL-LBR distribution in different cell lines (apart from the Hela cells that are presented in the main figures). Note that expression of the protein in U2OS cells causes major NE defects (arrow), as described previously by Zwerger et al. [34], whereas expression in all other lines has no visible effect. Bar, 6 μm. (TIF) [file pone.0169626.s001.tif]

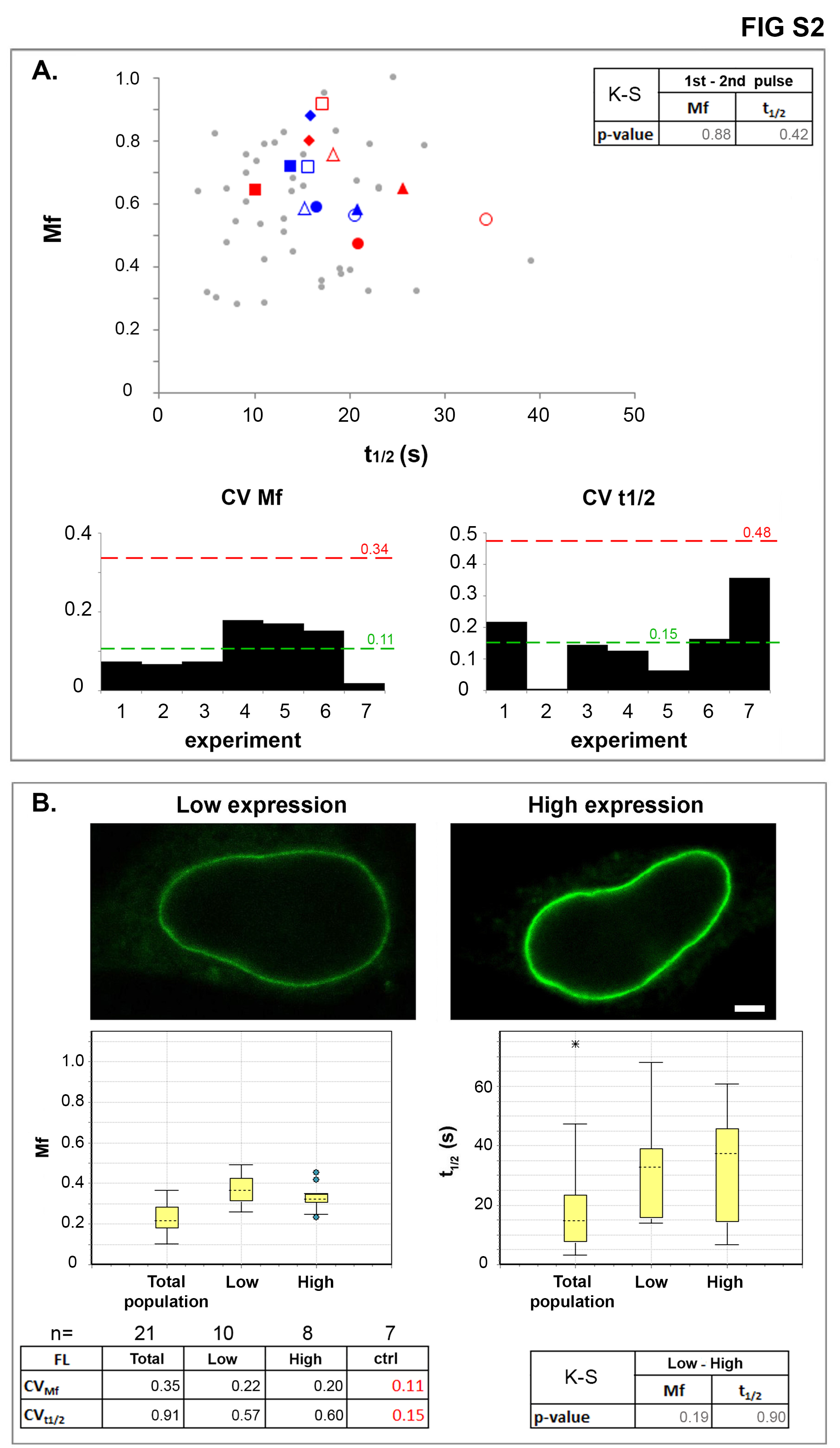

Supplement: S2 Fig — (A) Pulse-FRAP assays using an arc-shaped ROI configuration. The scatterplot shows the values of Mf and t1/2 in 7 independent experiments, where the same region of the NE was bleached and allowed to recover 2 times in a row with a 15-min interval in-between. The histograms underneath depict the CVs for Mf and t1/2. The first pulse in each sequence is represented by red symbols and the second by blue symbols. Grey symbols in the background correspond to the data shown in Fig 3 and are included for a comparison. The red (broken) line corresponds to the average CV calculated from the data shown in Fig 3; the green (broken) line corresponds to the average CV in the experiments presented here. The p-values of the K-S test (first versus second pulse) are indicated. (B) Protein mobility in cells expressing low and high levels of FL-LBR (see images; bar 6 μm). Mf and t1/2 box plots for all samples are presented. Statistical analysis using the K-S test, sample number and coefficients of variation (CVs) are specified. CVctrl indicates variation due to experimental error as determined in S2A Fig. (TIF) [file pone.0169626.s002.tif]

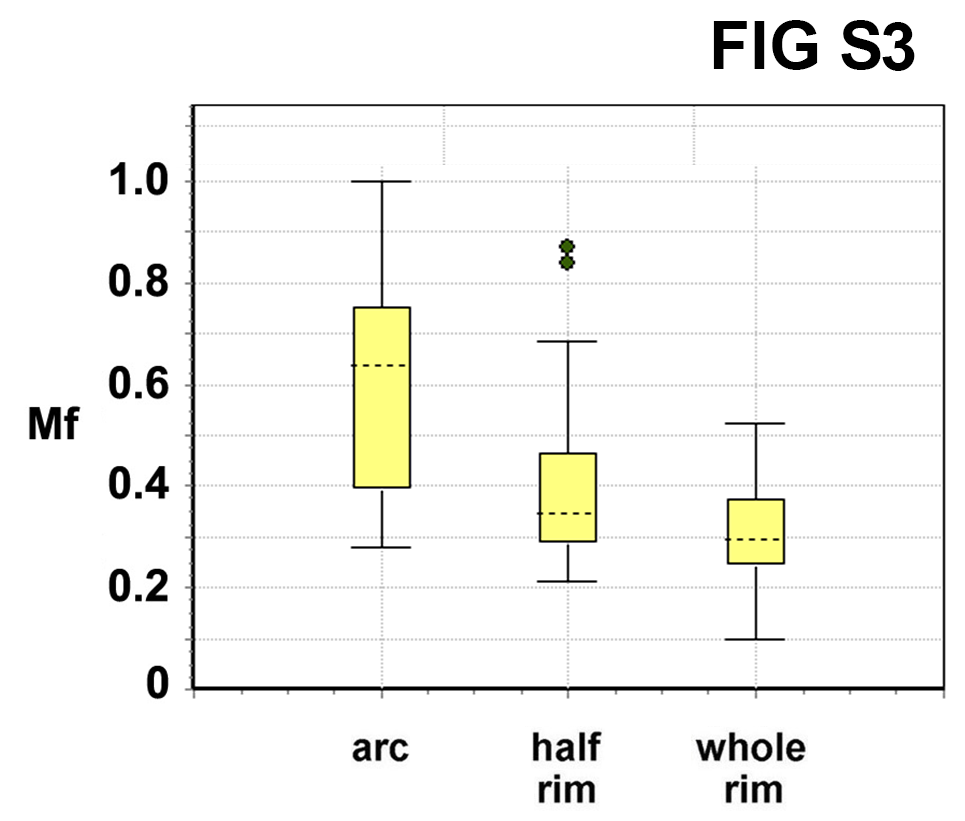

Supplement: S3 Fig — Mf box plot derived from FRAP assays using an arc-shaped ROI approximately 1.7 μm wide and 10 μm long, half-rim and whole-rim ROIs. (TIF) [file pone.0169626.s003.tif]

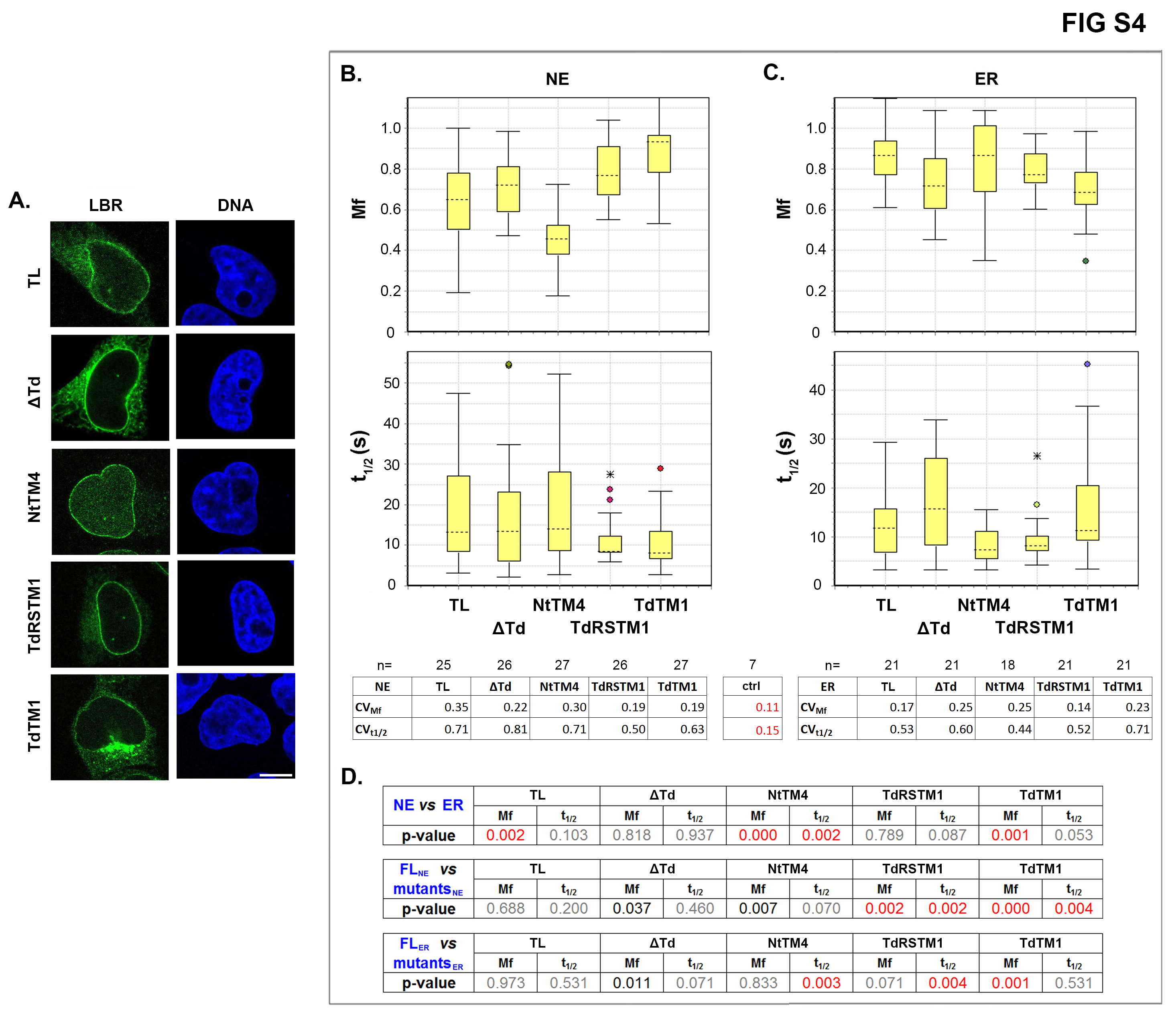

Supplement: S4 Fig — (A) Localization patterns of LBR mutants after transfection of Hela cells. Bars, 6 μm. (B-C) Mf and t1/2 box plots for all proteins using an arc-shaped ROI. The panel shows two separate sets of data obtained by probing either the NE or the peripheral ER. Sample number and coefficients of variation (CVs) are specified. CVctrl indicates variation due to experimental error as determined in S2A Fig. (D) Statistical evaluation and comparison of the data shown in (B-C) by the K-S test. DESCRIPTIVE TEXT: Dynamics of internally deleted and truncated LBR mutants To better understand the structure function relationships of LBR, apart from the mutants presented in the main text, we also examined the following mutants (S1A Fig): TL (missing the hydrophilic tailpiece); NtTM4 (missing the tailpiece and transmembrane domains I-III, V-VIII); ΔTd (missing the amino-terminal Tudor domain); TdRSTM1 (missing the tailpiece, the amino-terminal GD domain and transmembrane domains II-VIII) and TdTM1 (missing the amino-terminal RS and GD domains, as well as all sequences downstream to the transmembrane domain I). The subcellular distribution of these mutants was similar to that of FL-LBR (S4A Fig). However, when we assayed their diffusional mobility by FRAP and analyzed the data by the K-S test (S4D Fig), we arrived at some interesting conclusions. First, no statistically significant differences were found when we compared the mobility and diffusion rate of ΔTd and TdRSTMI in the NE and the bulk ER. This suggested that, unlike carboxy-terminal truncations, amino-terminal truncations affect severely the ability of LBR to bind to underlying sub-structure. Second, the TdRSTMI and the TdTMI mutants appeared to be more mobile and faster exchanging than FL-LBR at the NE. However, this was less apparent with the ΔTd mutant, although this (minimally truncated) protein was generally as mobile at the NE as was in the bulk ER (see above). When we attempted a similar comparison with the ER-distributed [file pone.0169626.s004.tif]

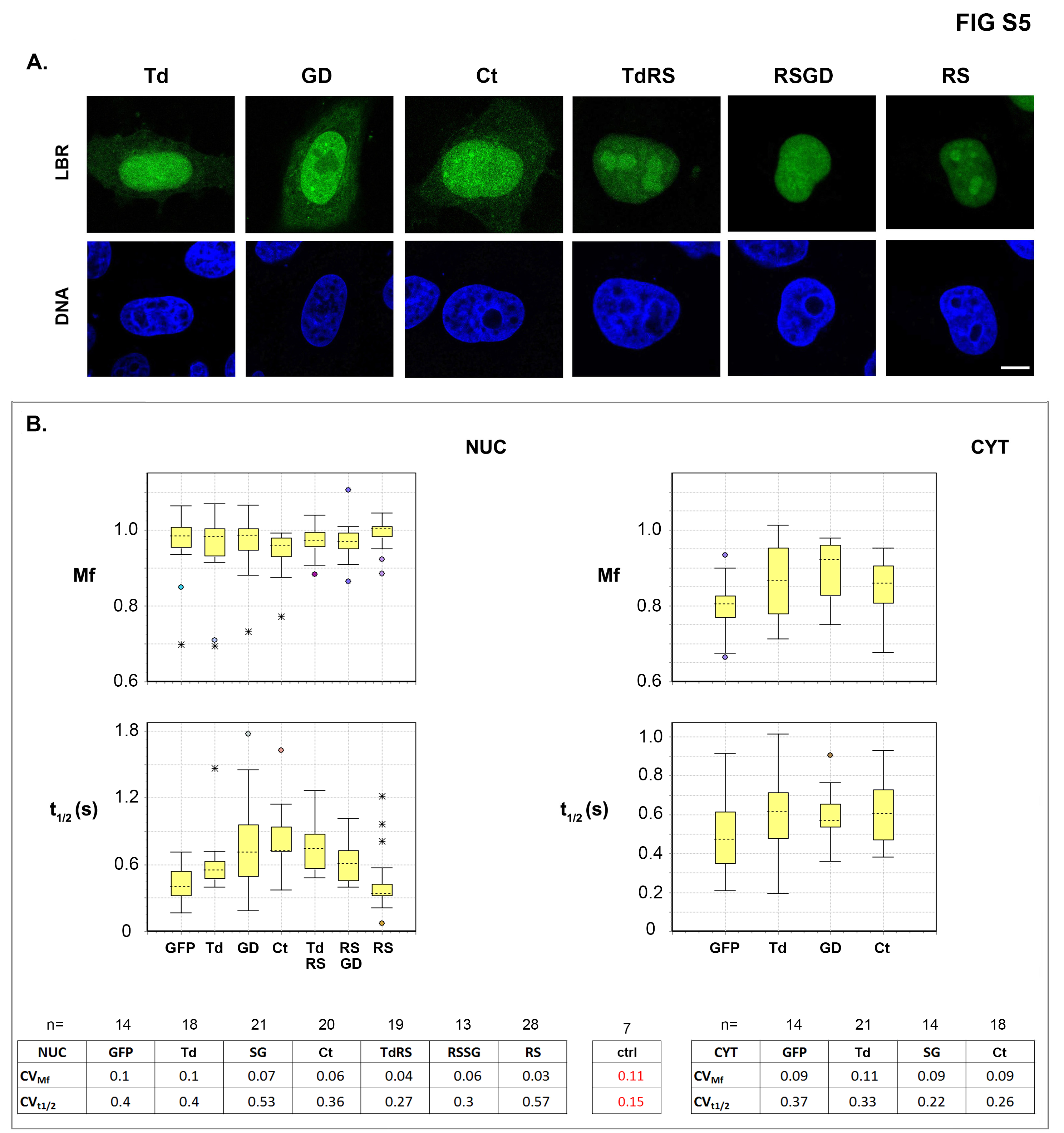

Supplement: S5 Fig — (A) Representative profiles of Hela cells expressing LBR mutants that do not possess a transmembrane domain (see S1A Fig). (B) Corresponding Mf and t1/2 box plots after assaying the mobility of the soluble mutants in the cytoplasm (CYT) or the nucleoplasm (NUC). Sample number and coefficients of variation (CVs) are specified. CVctrl indicates variation due to experimental error as determined in S2A Fig. (TIF) [file pone.0169626.s005.tif]
