# Supplementary material for: Dynamics and Structure-Function Relationships of the Lamin B Receptor (LBR)
Source: PLoS One. 2017 Jan 24;12(1):e0169626. doi: 10.1371/journal.pone.0169626 (PMC5261809; doi:10.1371/journal.pone.0169626)
Supplement: S1 Table — (DOCX) [file pone.0169626.s006.docx]

| **Supplementary TABLE I** | | |
| --- | --- | --- |
| **construct** | **Forward Primers** | **Reverse Primers** |
| **FL** | CCGGAATTCATGCCAAGTAGGAAGTTTGTTGAG | CGCGGATCCCTCTGTAAATGTAGGGGAATATGCG |
| **ΔTMV-VIII,CT** | CCGGAATTCATGCCAAGTAGGAAGTTTGTTGAG | CGCGGATCCCAGGGGCTCGCAGAGAGCGAA |
| **ΔTMVIII,CT** | CCGGAATTCATGCCAAGTAGGAAGTTTGTTGAG | CGCGGATCCCACTTTTCCCCGTGGAAGTATG |
| **ΔTD** | CCGGAATTCATGAGAAAAAGTGGCTCGATTTCCAGC | CGCGGATCCCTCTGTAAATGTAGGGGAATATGCG |
| **ΔCT** | CCGGAATTCATGCCAAGTAGGAAGTTTGTTGAG | CGCGGATCCCATGGATAAGCAAGGCGGTGAA |
| **ΔTMII-VIII,CT** | CCGGAATTCATGCCAAGTAGGAAGTTTGTTGAG | CGGGGATCCCAGCCGGCAGAGGTGGAG |
| **ΔTMI-III,V-VIII,CT** | CCGGAATTCATGCCAAGTAGGAAGTTTGTTGAG GAAGGACTTGGAGTACACTCACTTC | GAAGTGAGTGTACTCCAAGTCCTTC  CGCGGATCCCAGGGGCTCGCAGAGAGCGAA |
| **TDRSTMI** | CCGGAATTCATGCCAAGTAGGAAGTTTGTTGAG  GCTTCCCATTTTGGAGGAGTA | CTCCTCCAAAATGGGAAGCAG  CGGGGATCCCAGCCGGCAGAGGTGGAG |
| **TDTMI** | CCGGAATTCATGCCAAGTAGGAAGTTTGTTGAG  AAGCAATTTGGAGGAGTACCTGGTG | CTCCTCCAAATTGCTTAAAGG  CGGGGATCCCAGCCGGCAGAGGTGGAG |
| **TDRSGD** | CCGGAATTCATGCCAAGTAGGAAGTTTGTTGAG | CGCGGATCCCAGTGGTCACTTGAAAGGTTCC |
| **TDRS** | CCGGAATTCATGCCAAGTAGGAAGTTTGTTGAG | CGCGGATCCCATGGGAAGCAGAGACGGATC |
| **RSGD** | CCGGAATTCATGAGAAAAAGTGGCTCGATTTCCAGC | CGCGGATTCCCTCCAAGTCCTTCCTCTGTGGAGTGG |
| **TD** | CCGGAATTCATGCCAAGTAGGAAGTTTGTTGAG | CGCGGATCCCTTGCTTAAAGGATTTTAAAGGCTT |
| **RS** | CCGGAATTCATGAGAAAAAGTGGCTCGATTTCCAGC | CGCGGATCCCATGGGAAGCAGAGACGGATC |
| **GD** | CCGGAATTCATGGAGGGTGACGTGAAGGAGAAGAAGG | CGCGGATTCCCTCCAAGTCCTTCCTCTGTGGAGTGG |
| **CT** | CCGGAATTCATGCGAGAGGCCCGGGATGA | CGCGGATCCCTCTGTAAATGTAGGGGAATATGCG |
