# Supplementary material for: Dynamics and Structure-Function Relationships of the Lamin B Receptor (LBR)
Source: PLoS One. 2017 Jan 24;12(1):e0169626. doi: 10.1371/journal.pone.0169626 (PMC5261809; doi:10.1371/journal.pone.0169626)
Supplement: S1 Text — (DOCX) [file pone.0169626.s007.docx]

**S1 Text**

**Dynamics of internally deleted and truncated LBR mutants**

To better understand the structure function relationships of LBR, apart from the mutants presented in the main text, we also examined the following mutants (S1A Fig): TL (missing the hydrophilic tailpiece); NtTM4 (missing the tailpiece and transmembrane domains I-III, V-VIII); ΔTd (missing the amino-terminal Tudor domain); TdRSTM1 (missing the tailpiece, the amino-terminal GD domain and transmembrane domains II-VIII) and TdTM1 (missing the amino-terminal RS and GD domains, as well as all sequences downstream to the transmembrane domain I).

The subcellular distribution of these mutants was similar to that of FL-LBR (S4A Fig). However, when we assayed their diffusional mobility by FRAP and analyzed the data by the K-S test (S4D Fig), we arrived at some interesting conclusions. First, no statistically significant differences were found when we compared the mobility and diffusion rate of ΔTd and TdRSTMI in the NE and the bulk ER. This suggested that, unlike carboxy-terminal truncations, amino-terminal truncations affect severely the ability of LBR to bind to underlying sub-structure. Second, the TdRSTMI and the TdTMI mutants appeared to be more mobile and faster exchanging than FL-LBR at the NE. However, this was less apparent with the ΔTd mutant, although this (minimally truncated) protein was generally as mobile at the NE as was in the bulk ER (see above). When we attempted a similar comparison with the ER-distributed mutants, we did not find differences between TdRSTMI and ΔΤD, but TdTMI and ΔCt behaved differently in terms of mobility and exchange rate. From these data we infer that although amino-terminal truncations affect primarily LBR binding to the nuclear acceptor sites, the different mutant proteins do not diffuse with the same ease along the membrane.
